# Sensor-Based Optimization Model for Air Quality Improvement in Home IoT

**DOI:** 10.3390/s18040959

**Published:** 2018-03-23

**Authors:** Jonghyuk Kim, Hyunwoo Hwangbo

**Affiliations:** 1 Big Data Analytics Team, Kolon Benit Co., Ltd., 11 Kolon-ro, Gwacheon-si 13837, Gyeonggi-do, Korea; halfmoonlike@gmail.com; 2 Department of Data & Knowledge Service Engineering, Dankook University, 152, Jukjeon-ro, Suji-gu, Yongin-si 16890, Gyeonggi-do, Korea

**Keywords:** sensor-based home Internet of Things (IoT), indoor air quality, random data generation, spectrum/density analysis, natural cubic spline, user behavioral value

## Abstract

We introduce current home Internet of Things (IoT) technology and present research on its various forms and applications in real life. In addition, we describe IoT marketing strategies as well as specific modeling techniques for improving air quality, a key home IoT service. To this end, we summarize the latest research on sensor-based home IoT, studies on indoor air quality, and technical studies on random data generation. In addition, we develop an air quality improvement model that can be readily applied to the market by acquiring initial analytical data and building infrastructures using spectrum/density analysis and the natural cubic spline method. Accordingly, we generate related data based on user behavioral values. We integrate the logic into the existing home IoT system to enable users to easily access the system through the Web or mobile applications. We expect that the present introduction of a practical marketing application method will contribute to enhancing the expansion of the home IoT market.

## 1. Introduction

The home Internet of Things (IoT) is not entirely new. In the early 2000s, the widespread use of high-speed Internet and the wired Internet-based home network market rapidly expanded. The recent introduction of the home IoT is an extension of the existing market fostered by the development of the wireless Internet environment and machine to machine (M2M) technology [1]. While the existing home network has limitations in market expansion owing to the prevalent use of the wired network, the current home IoT can connect more diverse devices on account of the advancement of related telecommunication technologies. Accordingly, the current home IoT is distinguished from the existing home network and can be referred to as a new “ecosystem”.

The key features of the home IoT platform technology are summarized in Table 1. Nine core functions that should characterize a home IoT platform are listed.

In modern society, people spend more time indoors than outdoors. According to a World Health Organization study, people reside indoors for more than 21 h a day. The degree of indoor pollution varies by up to three times per individual, depending on the length of residence [7]. Indoor air is more polluted than outdoor air, which is naturally purified. However, it is not easy to recognize this condition and properly address it in real life. According to a US Environmental Protection Agency survey, the concentration of indoor air pollutants is two to five times, or even as much as 100 times, higher than outdoor air pollutants. It is well known that various kinds of volatile organic compounds (VOCs) that are harmful to humans are generated in indoor building materials, paints, and adhesives, which cause skin diseases and allergies [8]. As a practical example of indoor pollution, the health problem of “building syndrome” has emerged, with occupants complaining of temporary or chronic health problems relating to the building. We thus developed a model that can check the pollution of the indoor air in real time through a project conducted by “company A”. According to the model, the user is notified of the indoor air status and the appropriate ventilation time when necessary. The system is implemented to enable control from outside the building in real time by means of the user’s smartphone.

In this paper, we introduce the general procedure of the home IoT solution connected to a device sensor, IoT infrastructure, data processing, and mathematical modeling. We describe a related marketing strategy for the solution. In addition, we present specific modeling techniques for improving the air quality, which is a key home IoT service. The relatively sophisticated modeling technique is presented from an academic perspective. It is expected that the presented research will contribute to increasing the market integration of this type of solution by practical commercialization of models that can be readily applied.

The remainder of this paper is organized as follows. In Section 2, we summarize the smart home IoT system based on the user value and service vision along with research related to indoor air quality data processing and control systems. In Section 3, we describe our research design, which includes data collection and generation (scenario 1), and user-behavior settings with various statistical methodologies (scenario 2). We additionally introduce a marketing strategy for commercialization of home IoT technology. In Section 4, we conclude the paper and highlight the theoretical and practical implications of our research.

## 2. Background 

### 2.1. IoT and User Behavior Value

IoT is a system in which intelligent objects are connected in a physical or virtual space, and a network is formed between people and objects, or between objects and objects [9]. IoT can also be defined as a global infrastructure that provides intelligent services by combining knowledge based on context awareness. Implementation of an IoT requires an embedded system represented by things, a bi-directional communication environment, including the Internet, and commercial software to process the data.

IoT was initiated with the ability to remotely control lighting, thermostats, and security devices in everyday life [10]. This ability can be viewed as a function that satisfies user’s behavioral values (UBVs) of management, promptness, and information [11]. From that time, IoT has evolved into a means of exchanging information between objects and objects, and the “If This Then That” (IFTTT) concept has become universal, satisfying the value of scalability and automation. IFTTT represents a service for linking various programs and applications on the Internet with a computer through a command “recipe” [12]. In recent years, IoT in daily life has shown a tendency to expand its service centering on home IoT, which is fused with an artificial intelligence (AI) client. This enables users to manage multiple Internet devices more conveniently with voice commands.

In particular, a report summarizes existing high-level techniques in gas sensing and IoT-related papers published within the last five years. The research was tested in a kitchen environment that contained several objects monitored by different sensors [13]. The authors of the report introduced a representational and reasoning model for the interpretation of a gas sensor situated in the sensor network. The interpretation process includes inferring high-level explanations for changes detected over the gas signals. Inspired from the Semantic Sensor Network (SSN), the ontology used in this work provides an adaptive way of modeling the domain-related knowledge. Furthermore, exploiting Answer Set Programming (ASP) enables a declarative and automatic way of rule definition. Converting the ontology concepts and relations into ASP logic programs, the interpretation process defines a logic program whose answer sets are considered as eventual explanations for the detected changes in the gas sensor signals [14].

As the home IoT has become more convenient, it has become more widely used in everyday life. However, with this greater prevalence, users have become increasingly concerned about related privacy, security, and safety issues of home IoT devices. This concern is particularly the case with respect to the numerous sensors and communication devices involved. From the UBV perspective, IoT is demonstrating that the value placed on safety has recently increased along with universal UBV, such as manageability, speed, and scalability [11]. We derived 28 items on UBV based on the previous six years of IoT-related studies and theories of change. We redefine the three UBVs, as shown in Table 2, by incorporating the overlapping or similar concepts.

The theory of change emerged from the field of program theory and program evaluation in the mid-1990s as a new means of analyzing theories motivating programs and initiatives toward social and political change [19]. The theory of change generates knowledge about whether a program is effective, while explaining what methods the program can employ to be effective. In the early days of the theory of change, Kubisch established three quality control criteria to combine theory with traditional manufacturing, environmental psychology, organizational psychology, sociology, and political science [20]. The three criteria are plausibility, feasibility, and testability. Since the three criteria have been gradually extended to research on the theoretical background of system maintenance and software upgrades in information and communication technology, they have been used in various terms and as different values [21]. 

First, plausibility refers to the “logic of outcomes” pathway. In other words, it is the user’s expectation of or satisfaction with the accuracy and logic of the new technology in terms of UBV. Plausibility has been replaced by the meaning of relationship, sociality, and convenience in later studies. We redefine plausibility as interactivity by grasping the accuracy of the technique and the satisfaction of users accordingly. Second, feasibility refers to whether the initiative can realistically achieve its long-term outcomes and impacts. This has been handled in research in terms of the manageability of technologies to solve psychological problems related to the user’s reticent relation to the given technology. 

Thus, we contend that people using home IoT products or services can relinquish their technical reticence and gain psychological flexibility through certain values. We redefine all of these values as stabilities. Finally, testability refers chiefly to the indicator that measures the importance of users’ behavioral values. In other words, it is a type of instrumental utility that quantitatively measures thought flow and change. Recently, information and communication technologies (ICT) research has replaced testability with a kind of functionality. In this study, we redefine it as the comprehensive meaning of UBVs, such as scalability, compatibility, and promptness.

### 2.2. Studies on Improvement of Indoor Air Quality

A pleasant indoor environment is determined by the comprehensive action of various indoor environmental factors. In recent years, there has been a growing interest in indoor environmental factors that directly affect the degree of comfort for people who reside indoors, including temperature and humidity. In addition, there is a continuing need to manage indoor air quality factors, such as fine dust and carbon dioxide, which are closely related to human health [22].

According to US Environmental Protection Agency research, the causes of indoor hazardous substances are carbon dioxide (CO_2_), nitrogen dioxide (NO_2_), sulfur dioxide (SO_2_), ozone (O_3_), fine dust, heavy metal, asbestos, volatile organic compounds (VOCs), formaldehyde (H-CHO), microbial substances, and radon (Rn). Various gas measurement sensors for indoor air pollution sources have been developed and employed. Moreover, studies and development are currently underway on technologies that quickly detect flammable or toxic gases and respond accordingly [23].

Research on indoor air quality sensing has been conducted for various public places of everyday life, such as subways, schools, department stores, and offices. Paulos et al. [24] developed a system for measuring and monitoring office air quality through research on the office indoor air environment and work efficiency. As a result of controlling the system through a wireless sensor network linked to mobile devices, the overall work efficiency of the employees increased. Kanjo [25], Lohani and Acharya [26] developed their own environmental information monitoring system that applies precautions, such as indoor fine dust reduction, by using a mobile wireless LAN. The author showed that employee satisfaction with the work environment increased. Hwang and Yoe [27] monitored and analyzed indoor environment information through closed-circuit television (CCTV) and public environment information using an application programming interface (API). In addition, they developed an indoor environmental control system based on automatic situation recognition. Wang et al. [28] and Pötsch et al. [29] developed a wireless-sensor-based indoor environmental monitoring system for green buildings and the LoRaWAN stack, respectively. The system visualizes collected indoor environment data and measurement position data, and it distributes the temperature sensor to various locations in the target space. Moreover, it communicates the temperature in each space using a step color chart. Specifically, the authors calculated the distance from a window and installed sensors at three levels above the horizontal point. Their system visualizes the collected data as a three-dimensional space chart according to the spatial distribution. 

In a study on an indoor air quality monitoring system, researchers divided the measurement values of the fine dust concentration on the floor plan of the space into multiple spaces and expressed them in two or three dimensions [30]. The system has a simple structure for intuitively grasping the indoor environmental condition, thus enabling a comparison of the dust concentration according to the space. Meanwhile, the studies of Salamone et al. [31] utilize more simple self-developed experimental tools. They installed the open-source Smart Lamp in a real office environment and tested the reliability of IoT equipment. Salamone et al. [32] conducted a ventilation efficiency evaluation according to the ventilation method of an indoor space using a computational fluid dynamics (CFD) technique. To this end, they developed a system for measuring toluene concentrations and visualizing them in three-dimensional (3D) charts, which were applied to the field and contributed significantly to lowering the average toluene concentration.

Moreover, another paper presents a very important reference point on how to sense different kinds of gases. According to this study, the method of sensing various types of gas is described in detail. Additionally, the sensitivity (the minimum value of the target gas volume concentration when the gases could be detected) and the selectivity (the ability of gas sensors to identify a specific gas among a gas mixture) are regarded as very important measures for evaluating stability in gas sensing. In addition, it was explained that response time (the period from the time when gas concentration reaches a specific value to that when the sensor generates a warning signal), energy consumption, reversibility (whether the sensing materials could return to their original state after detection), adsorptive capacity, and fabrication cost are important factors.

As shown by the above research examples, most studies related to indoor air quality improvement involved developing a system that is suitable for a specific environment. This approach is difficult to apply to all environments of a given workplace using a standardized sensor device. Moreover, it cannot achieve the ultimate result needed for the actual user in the workplace, which is the reduction of harmful indoor components. In view of recent trends in the previous research, it can be observed that constructing the system environment that we planned, and creating the data through the distribution of the sample data, which is the methodology that is appropriate for it, is a very effective methodology. In other words, just as many experimental studies create experimental environments that can control variables themselves, we cannot only set specific situations, but we can also scientifically carry out all experimental steps consisting of system design, instrument connection, data communication control, sample data distribution analysis, and function estimation and verification. Many customized studies have been conducted through these actual system building processes [25,28,29,30,31,32], and the results are reflected very successfully in practice. From the researcher viewpoint, it is more effective to develop a system suitable for the environment and apply it to identifying problems and finding solutions. According to these trends, we intend to develop an air quality improvement system that can be applied to the apartment, the most common Korean housing type.

### 2.3. Technique of Random Data Generation

There are several ways in which we can amplify data within a given error-term. In particular, many previous studies on random number generation have been conducted based on the following three trends. First, in the information technology (IT) field, random number generation and its statistical evaluation have been mainly performed in the research of cryptography and system security. Second, prior research on random number generation in the financial sector has been predominantly focused on predicting how stock and bond values will change in response to changes in interest rates and other macroeconomic variables. Finally, another area that heavily uses random number generation is the traditional use of statistical tests to generate test data in areas where mathematical proofs are required.

Xiao et al. [33] argued that the most important point in generating test data is finding an efficient optimization algorithm. They generated test data using a genetic algorithm (GA), simulated annealing (SA), and genetic simulated annealing (GSA), and they concluded that GA is the best optimization algorithm for generating test data. Several studies were conducted to improve the efficiency of test data generation by improving existing optimization algorithms. Alba and Chicano [34] applied parallel GA to test data generation, and Mousa et al. [35] suggested application of a memetic algorithm that combines GA and local optimization algorithms. Watkins and Hufnagel [36] compared the fitness evaluation functions used to generate the test data. The results showed that the most efficient fitness evaluation functions for generating test data are BP1, BP2, and IPP.

Monte Carlo simulation (MCS) has been considered the most effective technique for random number generation for complex financial products. MCS is a common method that involves numerical integration based on random sampling. However, since random sampling is inherently a brute force method (BFM), many trials are required to maintain a high accuracy and minimum error rate, which is also time consuming. To solve this problem, Mallat [37] used the random number generation scheme (RNGS) to investigate bond values. This method stratifies sampling of interest rate data through a uniform distribution, applies an inverse-transform technique, and then obtains a random variable of an inverse function. The study of random sampling in the financial sector has centered on the interest rate structure; however, it has supplemented various alternative financial models, such as the standard Wiener process (SWP) [38]. In other words, a cumulative (or spectral or density) distribution function of the actual sampling data was converted into rich interest rate data and eventually the distribution function of the random data was generated through natural cubic spline (NCS) interpolation.

In this study, we employ Gerald and Wheatley’s random number generation method. We create a density distribution function based on the actual home IoT data, such as the indoor air quality concentration from apartment complexes and the API data provided by a meteorological office, and we extract the basic data. Based on these data, we generate a random number function for the last year of data through NCS interpolation. 

## 3. Design

### 3.1. Sensor-Based Modeling Framework

**Figure 1:**
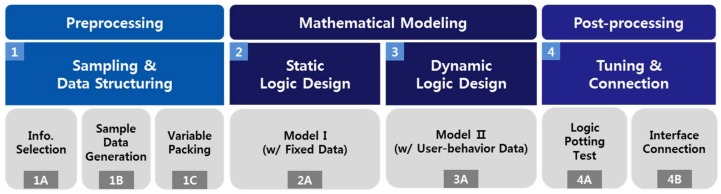
Modeling framework.

The model framework design for our study is divided into total three stages. First, as a preprocessing step, we select information, generate sample data, and pack it according to the time variable. The second step is the process of creating the model by building logic for the data. In this case, we proceed through two processes. First, we construct the model with the static data completed in the preprocessing process. Second, we construct the model through the variable data, such as the user behavior data. In the final step, post-processing, we evaluate the accuracy of the actual data with a continuous test, and we connect the constructed model to the existing interface. This sequence of steps is shown in Figure 1.

As mentioned earlier, we employ in this study the random number generation method presented in a previous study. We conduct a spectrum analysis based on the actual home IoT data and the public API data, and achieve a prototype of the sample data. We also apply the NCS interpolation method to the prototype data to generate a random number function for the last year of data.

### 3.2. Infrastructure 

We design the mobile application to transmit the information of each situation to the server so that users can collect IoT status information according to their situation. At this time, the managerial server that receives the user information simultaneously requests the status information of all the user’s home IoT devices, and it also structures and stores all the received information.

**Figure 2:**
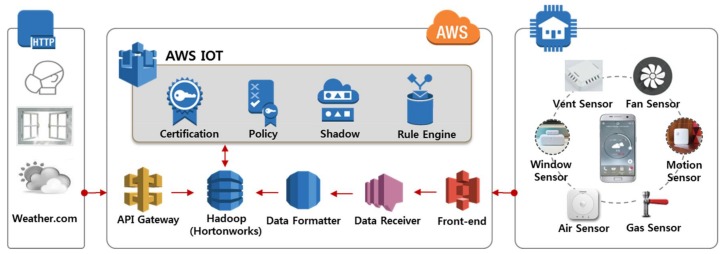
AWS-based home IoT platform.

The overall infrastructure is comprised of several components. First, the front-end receives the user’s status information from the mobile application and it helps the server structure and store data through its embedded business logic. Second, the client, acting as a data receiver, retrieves the change information of the user’s home IoT devices through the broker instance built into the system. It structures the data through the server’s business logic. Third, the IoT managerial connector, a module for communicating with the external server, manages the home IoT device information for each user. It also receives IoT device information and stores data at specific time intervals. Finally, the data formatter structures the state information of the user and the device proceeds through each module. This infrastructure is shown in Figure 2.

Each log data is structured and stored in Hadoop (Hortonworks), a data distribution storage processing framework. The document type can be divided into general data entered in the API, real user context data, and other data from connected home IoT devices. All of the IoT log collection servers that comprise this system are built in an Amazon Web Services (AWS) environment. Each component server constituting the system is composed as follows. First, we configure the log collecting Web server as an instant type (four CPUs, 8 GB memory, respectively). Second, in the case of the broker instance that transmits information of the IoT device, we construct an instance system by additionally connecting a 200-GB hard disk drive (HDD) to enable stable data transmission. Finally, in the case of Hadoop, which stores all user information, a 1-TB HDD is additionally connected to accommodate instantaneously changing data.

### 3.3. Preprocessing

After reviewing and evaluating as much information as possible about the air quality at the stage of variable selection, we identify the source of the relevant data, consider the possibility of analyzing the data, and finally select the variables. The selected variables are 21 in total. Among these, 12 outdoor data are retrieved via a public API, and indoor data are obtained from existing home IoT data. The results are shown in Table 3. In this study, statistical software packages such as SAS 9.4 (SAS Institute Inc., Cary, NC, USA), SAS Enterprise Miner v.13.1 (SAS Institute Inc., Cary, NC, USA) are applied to analyze the sensor data.

**Figure 3:**
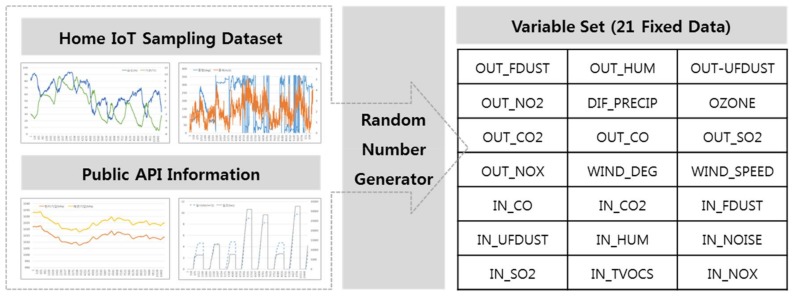
Random number generation.

As mentioned earlier, we visually check the temporal and seasonal flow of each variable and then apply the NCS interpolation method to each variable. In other words, we perform random number generation to fill each sample period consecutively in seconds for one year. This process is shown in Figure 3.

**Figure 4:**
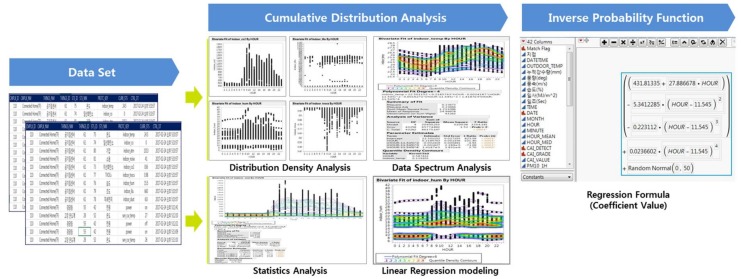
Cumulative distribution analysis.

In the next step, we replace the existing linear flow with a probability distribution function to make each variable value more fluid and objective. To this end, we use a cumulative distribution analysis (or density and spectrum analysis) method. We take an inverse function to express the probability function thus created as a variable coefficient value of one or less. Finally, we create a final data set for analysis by sorting the values of each variable into time variables in seconds and grouping them together. This process is shown in Figure 4.

### 3.4. First-Round Analysis

In the first round of the study, we strive to mathematically estimate and derive optimal indoor ventilation times to ensure the uncontaminated air quality. As a preliminary step, we estimate the environmental factors correlated with indoor air pollution, and we develop a model to derive air ventilation (*Vq*) and ventilation time (*Vt*) for optimal indoor air quality.

**Figure 5:**
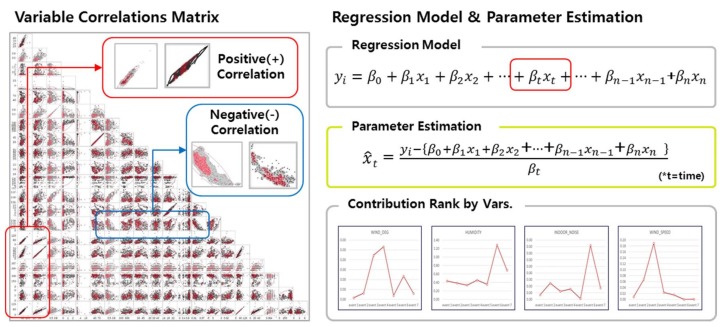
Analysis process in the first-round study.

The specific process is as outlined as follows. First, variables measured through the sensor are monitored to set an alarm when a certain threshold (pollution degree: 80%) of air pollution is exceeded. Secondly, we analyze pollutant variables that have the greatest impact on air pollution through correlation analysis of various air quality variables when an alarm occurs. Third, we compare indoor–outdoor observations of pollutant variables to determine whether indoor air is clean. Fourth, we predict the ventilation rate (*Vq*) by estimating the amount of the pollution factor. In this case, the amount of ventilation can be derived from the air pollution concentration minus the allowable pollution concentration as the denominator and the pollutant generation amount as the numerator. Finally, the optimal ventilation time (*Vt*) is estimated through the ventilation amount (*Vq*). This process is shown in Figure 5.

### 3.5. Second-Round Analysis

We create the UBV model by adding user-customized data in the second-round study, while creating the model for the existing fixed data in the first-round study. In other words, we add seven additional user variables to the existing 21 data items to estimate the optimal indoor ventilation time. This enables creation of a more subjective dataset with a range of predictions. Table 4 summarizes seven variables, which are classified into three categories: data from home IoT devices (4), data classified by a person’s characteristics (2), and three levels of place sizes (1).

**Figure 6:**
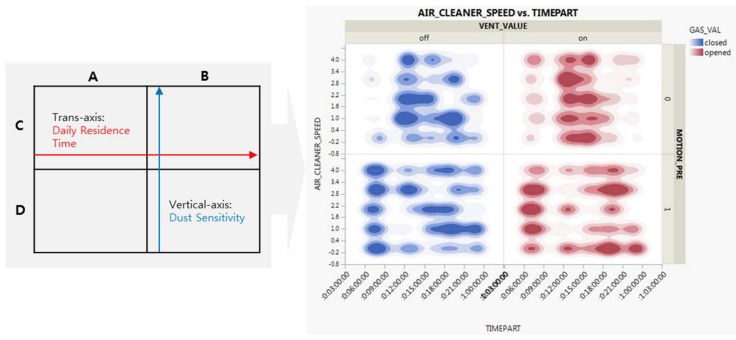
Four-dimensional clusters and variable distributions.

As shown in Figure 6, we obtain data in minutes from three sources. The first source is the home IoT device data stacked on the server. The second is a custom value we randomly group into four types. The last data source is the size of the place divided into three types. We create a new user-centric model by adding these three additional data items to the existing model. In other words, we intend to provide customized services for individual users. We thus design the set values into groups and develop a flexible logic according to the users. Technically, we construct new personal data for a total of 200 individuals, each consisting of 50 individuals in each of the four areas. The vertical axis denotes the residence time; the horizontal axis represents the user’s sensitivity to dust. Accordingly, we finally obtain a relational function model based on UBV.

### 3.6. Post-Processing

**Figure 7:**
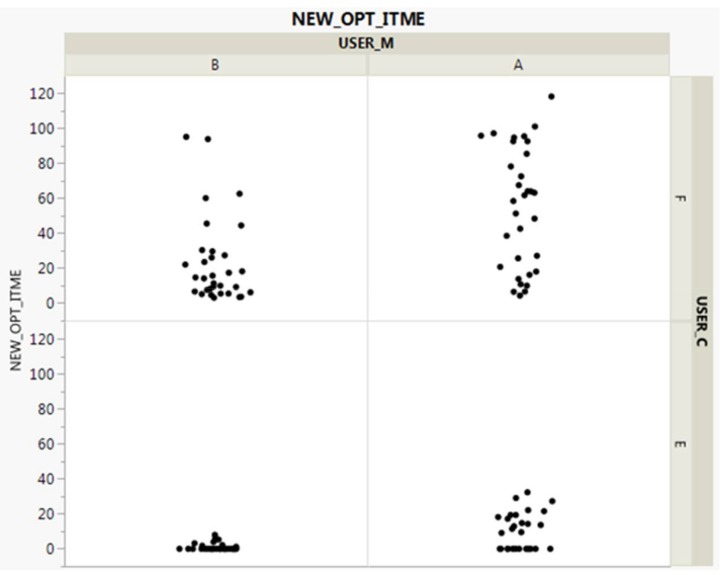
Model plotting test.

The most important goals of post-processing are summarized in the following two points. First, as shown in Figure 7, we realign and advance the logic through posterior conformance testing, which is a repeated visual plotting test of personalized data. Because our logic is automatic, regular, and dynamically generated for random numbers at specific locations and points in time, as well as for specific users, we believe it is necessary to stabilize them. Therefore, it is necessary to continuously check whether the value is within the error range under a certain condition while continuously visually confirming based on the newly generated random number.

**Figure 8:**
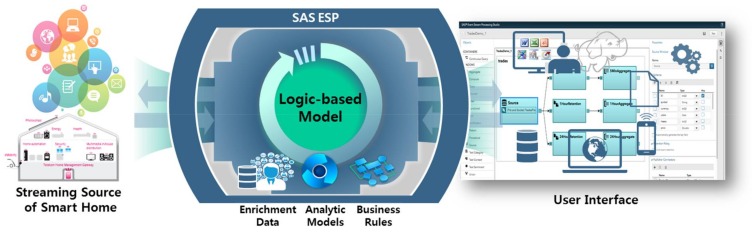
Connection to the user interface system by SAS ESP.

The second is to integrate all the created logic into the existing interface to enable users of the home IoT system via an existing PC or mobile device to actually observe the logic working. To this end, we used SAS Event Stream Processing (ESP; SAS Institute Inc., Cary, NC, USA), which provides streaming data of operations, transactions, sensors, and IoT devices in real time and visually presents them to the user. This process is depicted in Figure 8.

### 3.7. Marketing Prospects 

Depending on the use of the developed model in the business domain, we expect a notable adoption expansion in several markets. First, the model can be immediately applied to existing buildings as well as new buildings. This applied model will help to increase the market value of the building. That is, it can increase the value of existing products without requiring additional hardware or system changes. It is thus effective in terms of profit increases. In addition, the developed logic can be sold not only in the business-to-business market, but also as a business-to-consumer-specific product, by specializing it in a user-customized model. Second, by applying the latest data analysis model, we can expand brand awareness in the construction market. In maintaining the recent trend of the fourth industrial revolution, applying the latest IoT technology to the construction field can be expected to enhance the brand image in the home IoT market. In addition, we can expect to gain market dominance by supplying additional hardware and systems by selling artificially intelligent home IoT products, such as noise detectors and motion detectors, in line with rising brand awareness.

## 4. Conclusions

Recently, IoT has been used in a wide range of industries, including the smart home, health care, automobile, and energy industries. Many home IoT devices have already been integrated in our daily lives. In addition, IT-oriented companies and telecom companies that are leading IoT are expanding their market by developing technology-oriented products and services, while attempting to build a user-centered home IoT environment. Therefore, in this study, we conducted a literature survey on the user value of IoT based on the theme of air quality improvement among the IoT service examples. We focused on the user value that can be satisfied through related products and services. We also introduced random number generation as a method for reasonably amplifying analytical data, and created user-based analysis models through various data from smart home devices and public APIs. Moreover, we linked them to existing infrastructures. Finally, we described the use of the developed model for marketing purposes. 

## Figures and Tables

**Table 1:** Nine key features of the home IoT platform.

Function	Description	Prior Studies
Auto Configuration	Functions for device installation and easy configuration processing	Spanò et al. [2]
Remote Monitoring	Function to monitor human and object behavior according to space and time	
Situation Awareness	Function for real-time recognition of natural environment changes according to the situation	Alirezaie et al. [3]
Sensor-Driven Analytics	Function to support human decision-making through specific analysis and data visualization	
Process Optimization	Functions related to automatic control in specific environments, such as factories	
Energy Resource Optimization	Functions related to smart measurement and energy consumption optimization for energy (power, water, gas, heating, etc.) consumption	Sung and Chiang [4]
Privacy	Privacy protection function based on the user’s personal information, life patterns, and preference trends	Sicari et al. [5]
Open API	Support for managing multiple services, linking with external systems, and developing various “mashup” services	
Security	Function to ensure security against physical and logical intrusions	
Autonomous System	Functions for autonomous determination or automatic control of complex conditions	Gubbi et al. [6]

**Table 2:** Types of user behavioral value (UBV).

Redefined Factors of UBV	Operational Definition	Initial Factors of UBV	Prior Studies
Interactivity	Value in relation to the interaction with IoT devices	Objectivity, Completeness, Achievement, Logicality, Conductance, Accuracy, Satisfiability, Sociality, Expectancy, Relationship	Atzori et al. [15], Mennicken et al. [16]
Stability	Value for the manageability of IoT devices	Manageability, Simplicity, Safety, Security, Equity, Reliability, Transparency, Identity, Sustainability	Sicari, Rizzardi, Grieco and Coen-Porisini [5], Lee and Lee [11]
Functionality	Value for reliable operation of IoT devices	Convenience, Diversity, Compatibility, Scalability, Promptness, Efficiency, Informativeness, Automaticity, Usability	Kelly et al. [17], Vlacheas et al. [18]

**Table 3:** List of variables.

	Variable Name
Outdoor Information	Fine Dust ($\mu g/m^{3}$), Relative Humidity (%), Ultrafine Dust ($\mu g/m^{3}$), Nitrogen Dioxide (ppm), Precipitation (mm), Ozone Concentration (ppm), Carbon Dioxide (ppm), Carbon Monoxide (ppm), Sulfur Dioxide (ppm), Nitrogen Oxides (ppm), Wind Direction (8 dummy directions), Wind Velocity (m/s)
Indoor Information	Indoor Carbon Monoxide (ppm), Indoor Carbon Dioxide (ppm), Indoor Fine Dust ($\mu g/m^{3}$), Indoor Ultrafine Dust ($\mu g/m^{3}$), Indoor Relative Humidity (%), Indoor Noise (dB), Indoor Sulfur Dioxide (ppm), Indoor Volatile Substances (ppm), Indoor Nitrogen Oxide (ppm)

**Table 4:** Additional user-customized data.

Additional Data	Description
Device Data from IoT devices	Gas Valve Sensor (2 Levels, on/off)
	Ventilation Sensor (2 Levels, on/off)
	Air Cleaner Sensor (5 Levels, 0 for off and 1 to 4 for on)
	Movement Sensor (2 Levels, on/Off)
User Data	Dust Sensitivity (for Vertical Axis)
	Daily Residence Time (for Transverse Axis)
Space Size	3 Levels (60, 90, 120 Square Meters)
